# Compression-rate dependence of pressure-induced phase transitions in Bi

**DOI:** 10.1038/s41598-021-94260-y

**Published:** 2021-07-21

**Authors:** Rachel J. Husband, Earl F. O’Bannon, Hanns-Peter Liermann, Magnus J. Lipp, Alba S. J. Méndez, Zuzana Konôpková, Emma E. McBride, William J. Evans, Zsolt Jenei

**Affiliations:** 1 Deutsches Elektronen Synchrotron DESY, Notkestrasse 85, 22607 Hamburg, Germany; 2 High Pressure Physics Group, Lawrence Livermore National Lab, 7000 East Avenue, L-041, Livermore, CA 94550 USA; 3 Bayerisches Geoinstitut BGI, University of Bayreuth, 95440 Bayreuth, Germany; 4 European X-Ray Free-Electron Laser Facility GmbH, Holzkoppel 4, 22869 Schenefeld, Germany; 5 SLAC National Accelerator Laboratory, 2575 Sand Hill Road, Menlo Park, CA 94025 USA; 6 Stanford PULSE Institute, SLAC National Accelerator Laboratory, Menlo Park, CA 94025 USA

**Keywords:** Techniques and instrumentation, Condensed-matter physics, Phase transitions and critical phenomena

## Abstract

It is qualitatively well known that kinetics related to nucleation and growth can shift apparent phase boundaries from their equilibrium value. In this work, we have measured this effect in Bi using time-resolved X-ray diffraction with unprecedented 0.25 ms time resolution, accurately determining phase transition pressures at compression rates spanning five orders of magnitude (10^–2^–10^3^ GPa/s) using the dynamic diamond anvil cell. An over-pressurization of the Bi-III/Bi-V phase boundary is observed at fast compression rates for different sample types and stress states, and the largest over-pressurization that is observed is ΔP = 2.5 GPa. The work presented here paves the way for future studies of transition kinetics at previously inaccessible compression rates.

## Introduction

Bismuth, a high-Z metal that has a remarkably complex phase diagram at relatively low pressures and temperatures, has been extensively studied using conventional static and dynamic compression techniques. Recent time-resolved X-ray diffraction studies of shock- and ramp-compressed samples have revealed interesting compression-rate dependent phenomena such as shifts of phase transition boundaries^1^, the formation of metastable phases^2,3^, and the adoption of alternative phase-transforming pathways^2–4^. In order to identify the driving forces that result in deviations from the equilibrium phase diagram, it is necessary to understand the impact of phase transition kinetics over a wide range of compression rates. However, traditional dynamic compression (e.g. laser-driven and gas gun) experiments are performed at strain rates higher than 10^5^ s^−1^, which are over 5 orders of magnitude larger than those experienced during static compression. Consequently, the behavior of Bi at intermediate strain rates remains essentially unexplored. Access to this strain-rate regime is possible using piezo-driven dynamic diamond anvil cells (dDACs), which can generate compression rates up to 160 TPa/s (strain rates of ~ 10^2^ s^−1^)^5^. However, studies utilizing this technique are still scarce, with many relying on indirect methods of structural determination^6–10^. More recently, improvements in detector technology allow for the collection of time-resolved X-ray diffraction data from dynamically-compressed samples at kHz repetition rates^5^, which offers sufficient time resolution to accurately pinpoint phase transitions pressures up to ~ 1000 GPa/s.

Static compression studies reported Bi to transform to several different high-pressure polymorphs with well-characterized crystal structures on compression^11^. The monoclinic Bi-II structure has a narrow stability field (2.5–2.8 GPa), with Bi adopting the incommensurate ‘host–guest’ Bi-III structure between 2.8 and 7.7 GPa before transforming to the body-centered cubic (bcc) Bi-V at higher pressures^11^. A series of displacive atomic mechanisms have been proposed for structural pathways relating the high pressure polymorphs of Bi^12^; the reconstructive Bi-I/Bi-II transition was described as proceeding through a primitive cubic superstructure, whereas displacive mechanisms were proposed for the Bi-II/Bi-III and Bi-III/Bi-V transitions. However, these have not been experimentally verified or investigated using computational methods.

Early shock-compression studies saw evidence of phase transitions at pressures consistent with results from static compression studies^13–15^, although the methods used to identify these transitions did not yield any structural information. Ramp compression experiments reported a strain-rate dependence of the Bi-I/Bi-II phase boundary, exhibiting a significant shift to higher pressures above 5 × 10^6^ s^−1^ strain rates^1^. More recently, time-resolved X-ray diffraction experiments of dynamically-compressed Bi using XFELs and synchrotron techniques have allowed for phase-transformation pathways to be mapped with direct structural determination, revealing significant deviations from the equilibrium phase diagram that were not observed in earlier experiments^2–4^. Incommensurate Bi-III was not observed in any of these experiments on compression^2–4^, and instead Bi-V was observed alongside the unidentified metastable Bi-M at pressures as low as 3 GPa^2,3^. Although Bi-III was initially reported to form on shock release^4^, subsequent studies determined the Bi-V/Bi-I transition to proceed through the high temperature Bi-II’ phase^3^, or via the Bi-V/Bi-M/Bi-II/Bi-II′/Bi-I structural sequence^16^.

The absence of Bi-III in shock compression experiments raises the question of whether such complex host–guest structures can form on such short timescales; however, the observation of isostructural Sb-II during similar experiments on antimony^17^ suggests that this is not a general time-scale related phenomenon. The observation of Bi-V at lower pressures in shock experiments in comparison to static compression experiments is surprising, as kinetic hinderance typically results in an over-driving of the phase transition boundary^1^. Although kinetic hinderance at the lower-pressure Bi-II/Bi-III transition could result in the observation of Bi-II at higher pressures (in the stability field of Bi-III), it may alternatively result in the formation of an energetically-competitive phase such as Bi-M. However, the Sb-II/Sb-III transition (isostructural with Bi-III/Bi-V) is observed at significantly lower pressures on shock compression in comparison to static compression, raising the question of whether a negative pressure shift of the Bi-III/Bi-V phase line with increasing compression rate could explain the eventual disappearance of Bi-III under shock compression. Previous dDAC experiments reported an over-pressurization of the Bi-III/Bi-V phase boundary of ~ 2 GPa at ~ 180 GPa/s^18^. However, it is clear that their reported transition pressures are overestimated due to the limited time resolution offered by the PILATUS 2M detector used in the experiments (exposure time 90 ms with 10 ms readout), which is insufficient to precisely locate the phase boundary at compression rates higher than 10.0 GPa/s. The influence of compression-rate on the Bi-III/Bi-V transition at faster compression rates therefore remains an open question.

In this work, Bi has been compressed in the dDAC at different compression rates spanning five orders of magnitude (~ 0.01–780 GPa/s) to study the kinetics of the pressure-induced Bi-III/Bi-V phase transition for different sample forms (foil and powder). Experiments were performed on samples loaded with and without a pressure-transmitting medium (PTM), allowing the influence of the stress state to be evaluated. The structural behaviour of Bi was mapped out using time-resolved X-ray diffraction measurements with a maximum effective collection rate of 4 kHz, which were performed using the fast diffraction set-up at the Extreme Conditions Beamline (ECB, P02.2) at PETRA-III, Hamburg. Our Bi results are compared with those from previous laser-driven dynamic compression experiments, with the aim of providing a consistent description of dynamically-compressed Bi in these different strain-rate regimes.

## Results

### Static compression experiments

**Figure 1:**
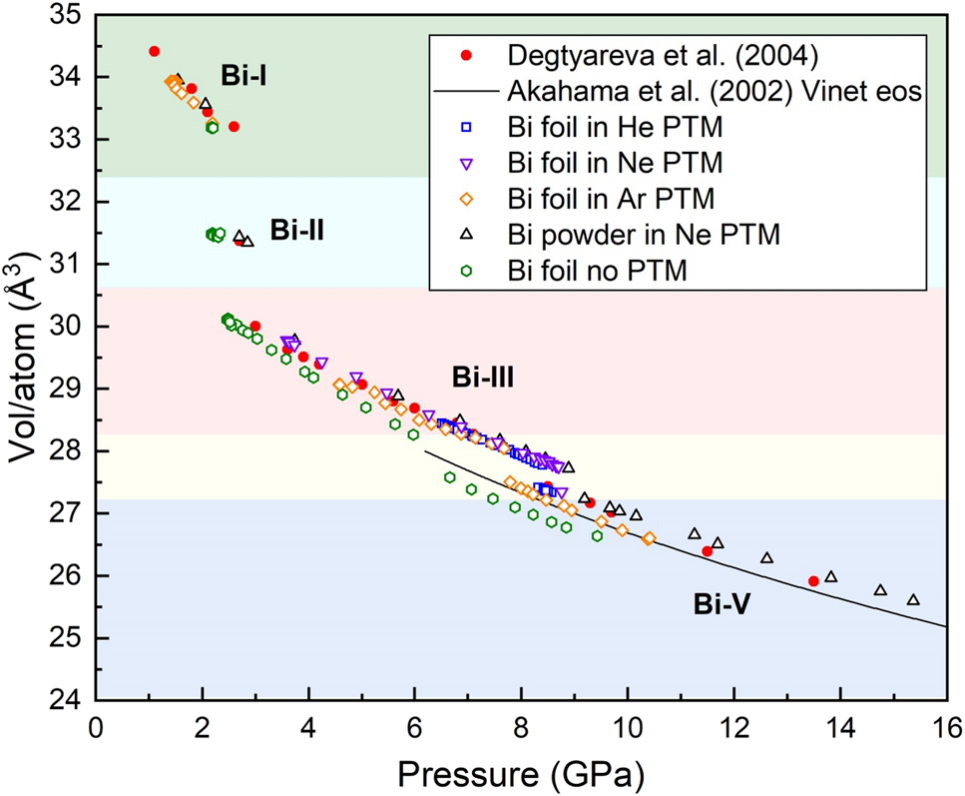
Volume/atom of Bi as a function of pressure for static compression experiments. Data were collected from Bi foil samples loaded in He, Ne, Ar, and without a PTM, and a powder sample loaded in Ne. The foil sample loaded without a PTM was prepared with the Au placed between the Bi and the diamond anvil. These data are compared with data from Degtyreva et al.^11^ and the Vinet EOS for Bi-V from Akahama et al.^21^.

Although the structural phase transitions of Bi and their associated transition pressures have been well documented, there is a surprising lack of consensus over the location of the Bi-III/Bi-V phase boundary, with reported values varying by as much as 2.65 GPa^19,20^. For this reason, we performed a series of static compression experiments to determine the Bi-III/Bi-V phase boundary for different loading conditions (Fig. 1), which allows for a direct comparison with results from our dDAC experiments.

Overall, a higher Bi-III/Bi-V transition pressure is observed in the quasi-hydrostatic samples (loaded in Ne and He) compared to the sample loaded in Ar, with an even lower transition pressure observed in the sample loaded without a PTM (Table 1). The compression curves of samples loaded in He and Ne are in good agreement with data from Degtyareva et al.^11^, whereas the data from the Ar sample show a higher compressibility in the Bi-V phase and are in good agreement with the Vinet EOS reported by Akahama et al.^21^ which was collected from samples loaded without a PTM.

**Table 1:** Bi-III/Bi-V transition pressures for samples compressed in a range of different pressure-transmitting media.

Sample type	Pressure medium	Pressure determinant	Transition pressure (GPa)
Powder	Ne	Au	9.04 (15)
		Bi	9.03
Foil	Ne	Au	8.73 (2)
		Bi	8.64
Foil	He	Au	8.42 (2)
		Bi	8.53
Foil	Ar	Au	7.74 (6)
		Bi	8.10

For samples loaded without a PTM, the use of an internal standard was found to be an unreliable method of pressure determination (Fig. S1), particularly when samples are dynamically-compressed (Figs. S2, S3, S4). For this reason, we have chosen to report transition pressures based on the unit cell volume of Bi-V, which is a true description of the sample state. Unless stated otherwise, all transition pressures reported in the remainder of the manuscript are determined using this method. Despite the limitations of Au as an internal pressure standard (for samples loaded without a PTM), all samples in the dDAC experiments were loaded with Au as a convenient method of tracking the approximate pressure through the compression cycle, as Au remains fcc across the entire pressure range.

### dDAC experiments: With a pressure-transmitting medium

**Figure 2:**
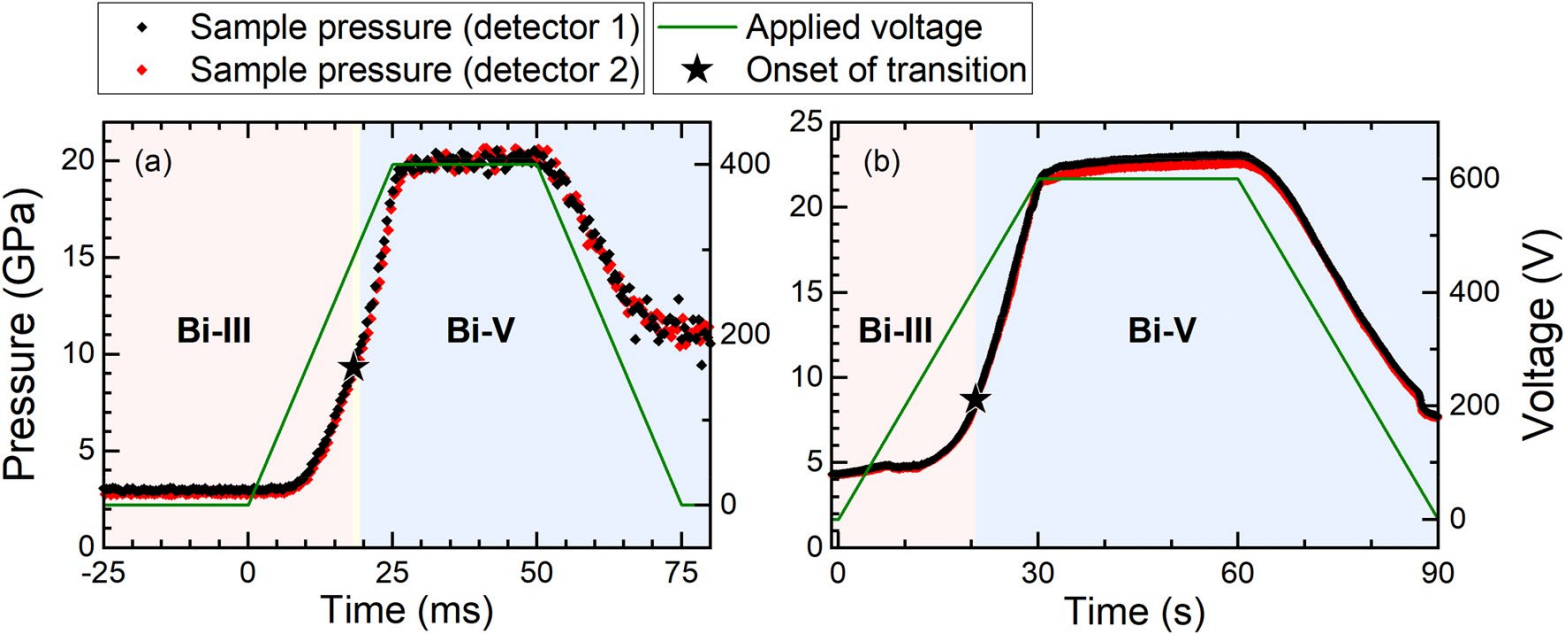
Time-dependent pressure profile for (**a**) fast and (**b**) slow compression cycles on Bi foil using the dDAC, and the corresponding voltage applied to the piezo actuator. The pressure was estimated from the Au pressure marker, as described in the text. The stars indicate the onset of the Bi-III/Bi-V transition. The average compression rates during the ramp are (**a**) 680 and (**b**) 0.56 GPa/s and the instantaneous compression rates at the transition are (**a**) 785 and (**b**) 1.13 GPa/s.

**Figure 3:**
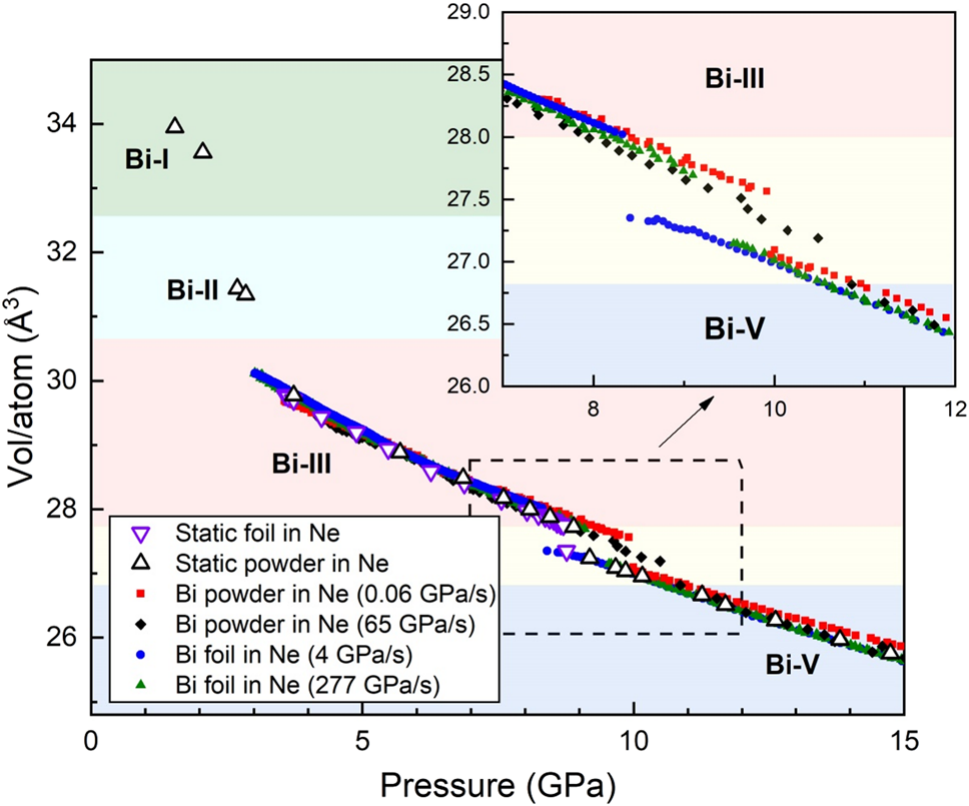
Volume/atom as a function of pressure for selected dDAC compression ramps on Bi samples loaded with Ne. Data from fast and slow dDAC compressions of both Bi foil and powder samples are compared with the results from static compression experiments on Bi powder and foil samples loaded in Ne from Fig. 1. The inset shows an enlargement of the ramp data in the vicinity of the Bi-III/Bi-V transition to highlight the different transition pressures. The pressure was estimated from the Au pressure standard as described in the text.

To investigate the compression rate dependence of the Bi-III/Bi-V phase boundary under quasi-hydrostatic compression, experiments were performed on Bi foil and powder samples loaded with a Ne PTM. Examples of typical time-dependent pressure profiles for foil samples are shown in Fig. 2, and compression curves for selected fast and slow compression ramps on foil and powder samples are shown in Fig. 3. The dDAC compression curves are in good agreement with those from our static compression experiments on samples loaded with Ne, indicating that Bi and Au are under the same stress state. For both sample types, a higher transition pressure is observed in the fast compression experiment compared to the slow compression (ΔP = 0.6 GPa for the foil and 1.0 GPa for the powder), and the powder samples have consistently higher transition pressures than the foil.

**Figure 4:**
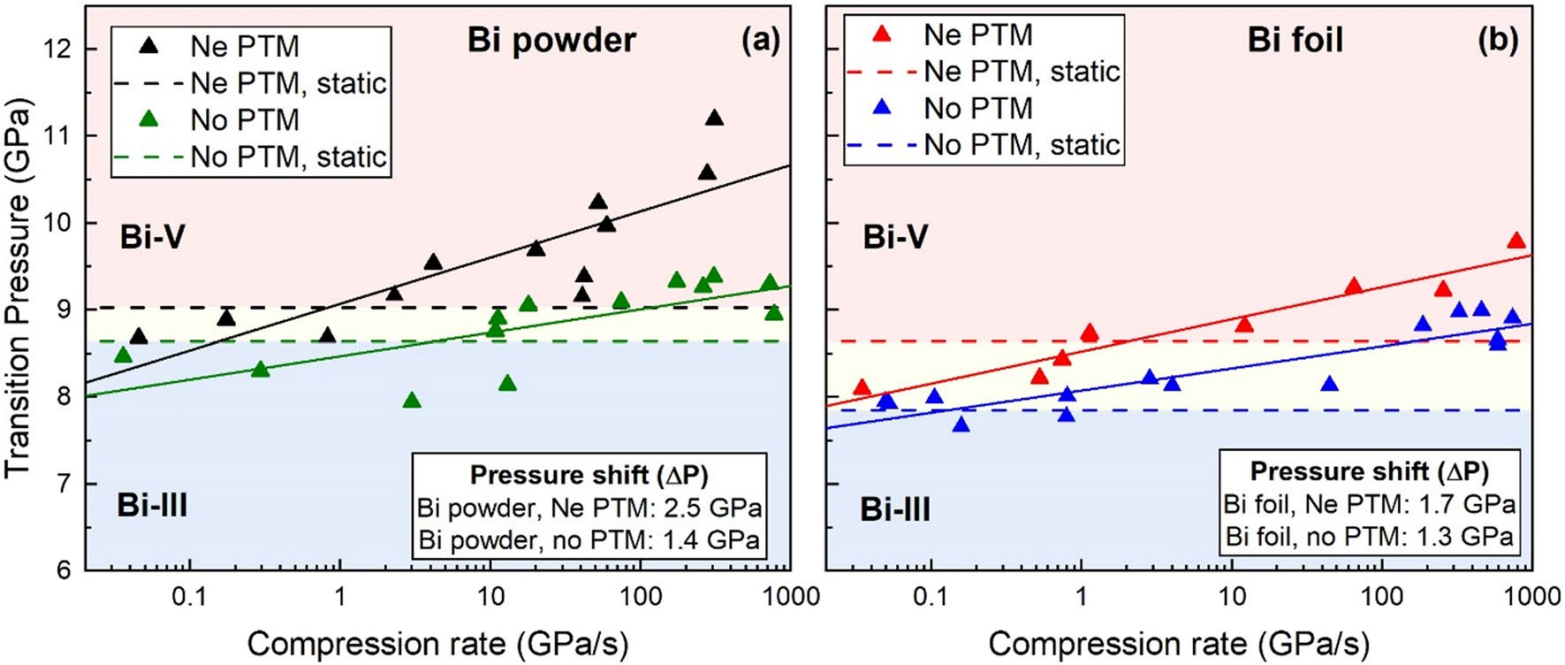
Bi-III/Bi-V transition pressure as a function of compression rate for different sample types (foil and powder) and different sample conditions (with and without a PTM). The transition is identified by the first appearance of Bi-V in the diffraction patterns, as described in the text. The solid lines show a fit of the dynamic compression data to functional form $y=Alog\left(x\right)+B$, and the resultant fit parameters are given in Table S2.

A summary of the Bi-III/Bi-V transition pressures as a function of compression rate for all samples is shown in Fig. 4, where a clear compression-rate dependent trend is observed for both powder and foil samples. At low compression rates, the transition is observed at slightly lower pressures than in the static compression experiments with a Ne PTM, whereas at fast compression rates the transition is shifted to higher pressures. Compression rates generated by the dDAC are more controlled than those in membrane-driven static compression experiments, in which the instantaneous compression rate at the Bi-III/Bi-V transition may be higher than in the slow dDAC compressions, which may explain the observation of lower transition pressures at low compression rates in comparison to static compression experiments. In addition, the density of data points collected in slow dDAC experiments is significantly higher than in the static compression experiments, which allows for the transition pressure to be determined with a higher precision. A larger compression-rate dependent shift is observed for the powder samples (ΔP = 2.5 GPa) compared to the foil samples (ΔP = 1.7 GPa.). In order to illustrate the changes of the transition pressure as a function of compression rate, the data collected during dynamic compression were fit to the functional form $y=Alog\left(x\right)+B$, and resulting fit parameters are given in Table S2. The data could also be fit assuming a critical compression rate of 1 GPa/s (Fig. S5); however, the small number of data points at low compression rates and the scatter in the data make it difficult to draw any firm conclusions.

### dDAC experiments: without a pressure-transmitting medium

**Figure 5:**
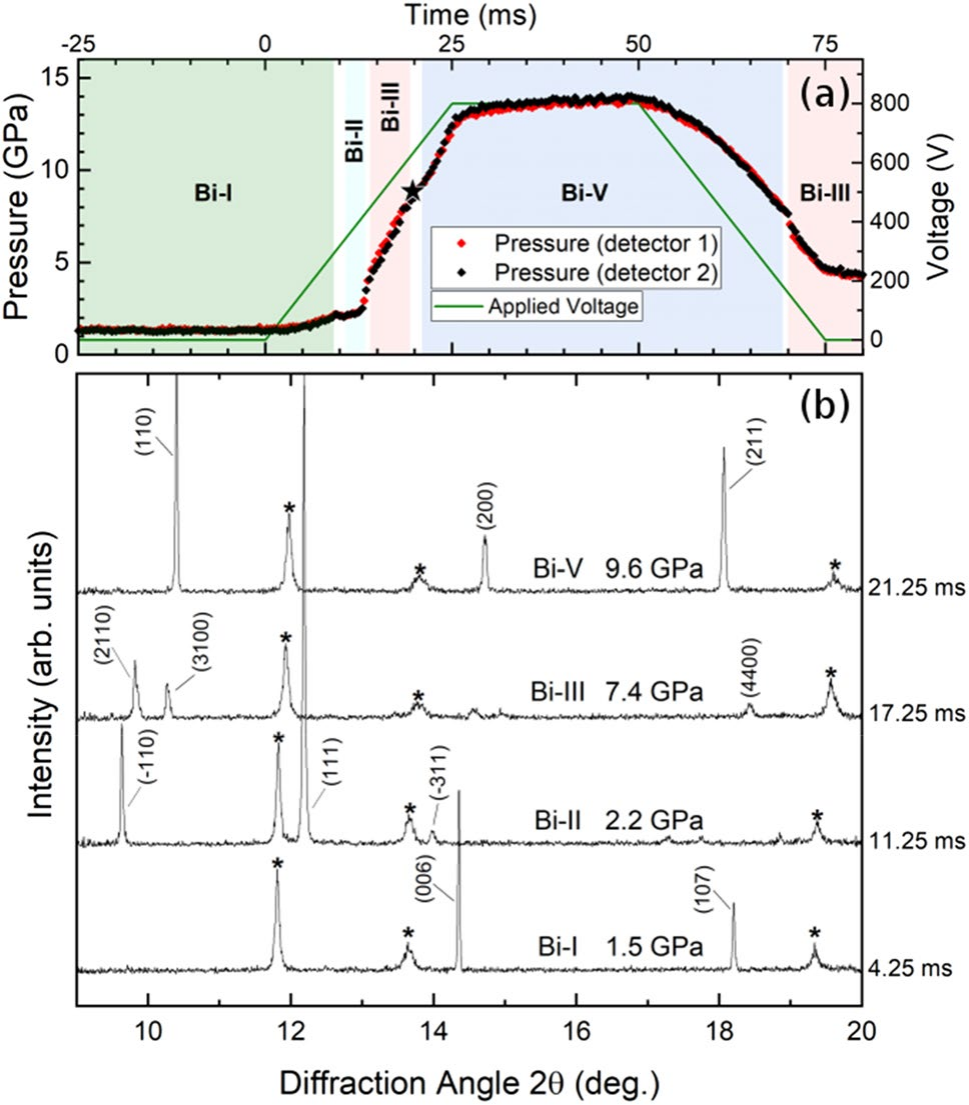
(**a**) Compression profile, applied voltage and (**b**) integrated X-Ray diffraction profiles from a 25 ms compression ramp of a Bi foil sample without a PTM. Data were collected using a 0.5 ms exposure time. The shaded regions in (**a**) indicate regions in which single-phase Bi patterns were observed, and the star indicates the first appearance of Bi-V. Although not all of the Bi Bragg reflections are observed due to preferred orientation of the highly textured Bi foil, the different high-pressure phases are easily identified due to the emergence of new peaks at the phase transition. In (**b**), Bi reflections are labelled with their Miller indices, and peaks from the Au pressure marker are marked with an asterisk. The pressure is determined from Au, which is known to underestimate the sample pressure in this loading configuration (Au between the Bi sample and the diamond anvil, see Supplementary Methods).

Dynamic compression experiments were also performed on Bi foil and powder samples loaded without a PTM. These results can then be compared with those from the previous section in order to investigate the compression-rate dependence of the Bi-III/Bi-V transition pressure under different stress conditions (Fig. 4), as a larger degree of deviatoric stress is present in samples loaded without a PTM in comparison to those loaded in Ne (Fig. S6). An example of a typical time-dependent pressure profile and integrated diffraction patterns for a 25 ms compression of a Bi foil sample are shown in Fig. 5; although not all Bragg reflections were observed due to preferred orientation in the Bi sample, phase transitions can be easily identified by the appearance of new peaks which belong to the higher pressure phase.

A clear compression-rate dependent trend is observed in the foil samples (Fig. 4b), where the transition pressure is shifted to higher pressures at fast compression rates. The phase boundary displays a similar compression rate dependence to that observed in the foil samples loaded in Ne but at lower pressures (ΔP = 1.7 GPa for samples in Ne and 1.3 GPa for samples loaded without a PTM). The data from powder samples (Fig. 4a) loaded without a PTM are more scattered, and the trend with compression rate is not as clear. Our fit to these data reveal that there is an increase in the transition pressure as compression rate increases. A much smaller compression-rate dependent shift is observed for the powder samples loaded without a PTM (ΔP = 1.4 GPa) in comparison to powder samples in Ne (ΔP = 2.5 GPa).

The results shown in Fig. 4 include multiple data points from the same sample i.e. the sample was compressed multiple times through the Bi-III/Bi-V transition at different rates to collect multiple data points. This introduces the possibility that microscopic changes in the sample material such as defect generation could influence the transition pressure in subsequent compressions. The influence of multiple compressions across the Bi-III/Bi-V transition was therefore investigated by monitoring the transition pressure during pressure cycling (Fig. S7a,b). The measured transition pressure of Bi is constant as a function of compression cycle, and is in excellent agreement with results from newly loaded samples compressed with the same rise time (Fig. S7c). These results are a strong indication that we are observing a compression rate dependent phenomenon in our dynamic experiments, rather than the hindering or facilitation of the transition due to dislocation formation.

## Discussion

Our results reveal an over-pressurization of the Bi-III/Bi-V phase boundary at fast compression rates for different sample types (powder and foil) and stress states (with and without a PTM). The largest over-pressurization is observed for the powder samples loaded in Ne (ΔP = 2.5 GPa); we therefore considered the possibility that over-pressurization is enhanced by the smaller grain size of the powder sample in comparison to the foil. However, static compression experiments on Bi nanoparticles have shown that grain size does not influence the Bi-III/Bi-V transition pressure until particles sizes below ~ 250 nm^20^, suggesting that our observations cannot be explained by size effects alone. This is supported by our observation that transition pressures for powder samples loaded without a PTM are in good agreement with those from foil samples. We also considered the possibility that the observation of lower transition pressures in the foil samples compared to the powder could be attributed to the stress state of the sample; in the powder, individual grains are surrounded by Ne, whereas only the bulk material is surrounded by Ne in the foil samples. The powder sample may therefore have lower differential stress than the foil. However, a comparison of results from the foil samples loaded with and without a PTM suggests that non-hydrostatic stress only has a minor effect on the observed over-pressurization.

We therefore attribute the observed differences between the powder and foil samples to our ability to detect the first traces of Bi-V in the diffraction patterns. The powder sample has a larger surface area and consequently a higher density of defects that act as nucleation sites, whereas the foil has a smaller surface and fewer defects. Consequently, the diffraction rings from the different sample types have very different microstructure; the large number of randomly-orientated crystallites of Bi-V in the powder distributes the intensity over the entire Debye ring, whereas the foil sample produced arcs and single-crystal like peaks that are present in just a small fraction of the ring (Fig. S8). The single-crystal like peaks and arcs concentrate the diffracted intensity onto one spot on the detector, enhancing the signal-to-noise ratio in comparison with the relatively-uniform intensity distribution of the powder. This is more pronounced for samples loaded in Ne, due to the smaller sample sizes in comparison to those loaded without a PTM.

Multiple factors can play a part in the observation of rate-dependent transition pressures. Firstly, it is possible that an insufficient volume of Bi-V has formed during the short timescales involved in the fast compressions to be detected by the X-ray diffraction probe, most notably at the short exposure times required for the fastest compression experiments. However, this cannot explain the observation of higher transition pressures in the powder samples, which have a higher density of nucleation sites. It is also possible that Bi-V does not form until higher pressures due to kinetic hindrance^22^, which could explain our observations. Therefore, although previous work has interpreted the proposed displacive mechanism to mean that kinetic hindrance should not be significant^3,4^, this is not consistent with our observations. Although this transition mechanism was determined to be a unique solution corresponding to the minimal energy pathway (when geometrical and symmetry arguments are considered), the proposed atomic displacements may still be associated with a significant energy barrier. In particular, a similar displacive mechanism proposed for the fcc-incommensurate transition in K was shown to have impossibly high kinetic barriers^23^, where instead it was shown to be more energetically favorable for the transition to proceed through a transient amorphous state. For a more comprehensive analysis of the kinetics of the phase transition, the Bi-III/Bi-V volume fractions should ideally be analyzed using the Johnson–Mehl–Avrami–Kolmogorov equation to account for the pressure-dependence of kinetic parameters such as activation energy, nucleation and growth rates^24^, which would allow for grain size or stress effects to be assessed. Unfortunately, the single-crystal-like nature of the diffraction patterns makes it very difficult to accurately determine phase fractions. We hope that these results will stimulate theoretical studies on the Bi high-pressure phase transitions to investigate the phase transition mechanisms and their associated kinetic energy barriers.

The observed over-pressurization at rapid compression rates should also be compared to the results from laser-driven shock compression studies, with the aim of forming a coherent description of the behaviour of Bi over a wide range of strain-rates. Bi-III is not observed in shock compression studies, and Bi-V is first recorded as low as ~ 3 GPa alongside Bi-M^2,3^. One possible explanation for this could be a negative pressure shift of the Bi-III/Bi-V phase boundary under fast compression, which would be consistent with observations relating to the Sb-II/Sb-III (isostructural with Bi-III/Bi-V) phase boundary under shock compression^17^. However, this is not consistent with our observations, which find an increase in the stability field of Bi-III as a function of compression rate. Differences between results from shock and static compression studies can also be related to sheer stresses; static compression experiments aim to be hydrostatic, whereas shock compression is uniaxial. For example, strain-rate lowering of two subsequent phase transitions in shock-compressed Si was attributed to shear stresses resulting from the anisotropic nature of shock compression^25^. It is therefore possible that shear stresses associated with shock compression facilitate the transition to Bi-V. However, the results from our foil samples compressed with and without a pressure transmitting medium suggest that the stress state has a relatively minor effect on the observed over-pressurization of the phase boundary. In order to investigate whether the absence of Bi-III under shock compression is related to the Bi-II/Bi-III phase transition pathway, future work should focus on the effects of compression-rate and stress state on the Bi-II/Bi-III transition, with the aim of understanding the structural mechanism and driving force that results in the formation of Bi-M.

We note that laser-driven ramp (quasi-isentropic) compression experiments did not observe shifts of the Bi-I/Bi-II phase boundary until strain rates above 5 × 10^6^ s^−1^^1^. The observation of Bi-V at pressures as low as 3 GPa in shock experiments suggest that the stability field of Bi-II is also relatively insensitive to high strain rates (Bi-II is stable in the 2.5–2.8 GPa pressure range under static compression), and so phase boundary shifts will most likely be much smaller than we observe for the Bi-III/Bi-V transition (ΔP = 2.5 GPa). It is therefore surprising that we observe similar behaviour at the Bi-III/Bi-V phase boundary at strain rates that are orders of magnitude lower (~ 10 s^−1^) than those in the ramp compression experiments. The observation of kinetic effects at the relatively slow compression rates experienced in this study suggest that care should be taken when interpreting experimental results from shock or dDAC compression studies for materials that undergo structural phase transitions. In particular, when results from shock compression experiments are used for modelling of ‘static’ processes (i.e. planetary interior conditions) for extreme P–T states that cannot be accessed using static compression methods, kinetic effects cannot be ruled out.

These X-ray diffraction experiments are at the forefront of what is possible for time-resolved dDAC experiments at 3^rd^ generation synchrotron light sources. Achievable time resolution is constrained by both the available X-ray flux and detector frame rate. Future upgrades to diffraction-limited storage rings such as PETRA-IV should allow for similar experiments to be extended to lighter elements. Moreover, faster detectors combined with hard X-ray Free Electron Lasers should extend dDAC studies to compression rates beyond 1000 GPa/s.

## Methods

### Samples: static compression

Static compression experiments were performed using membrane-driven DACs equipped with standard design diamond anvils with culet sizes ranging from 200 to 750 μm and stainless-steel gaskets. Two different Bi sample types were used. For some experiments, a small piece of Bi was cut from a larger piece of Bi foil (99.999% purity from Alfa-Aesar) and loaded directly into the sample chamber. For other experiments, Bi (99.5% purity with an average grain size of 44 μm from Alfa-Aesar) was ground into a powder before loading. For samples loaded with a PTM, DACs were loaded with a Ne, He, or Ar PTM using the in-house gas loading system at the ECB or at LLNL, where care was taken to ensure that the sample did not bridge between the opposing diamond anvils. Unless specified, Au powder (99.96% pure spherical powder from Alfa-Aesar with a grain size ranging from 0.8 to 1.5 μm) was included as an internal standard and the pressure was determined using the equation of state by Fei et al.^26^. The samples loaded with a PTM included a small grain of Au powder next to the Bi so that both materials could be illuminated by the X-ray beam. For the samples loaded without a PTM, the location of the Au within the sample chamber is specified in the text. One sample was prepared with a piece of Cu foil (99.97% pure 2 µm thick foil from Goodfellow) placed between the Bi foil and the diamond anvil, and the pressure was subsequently determined from the Cu EOS by Dewaele et al.^27^. The Bi-V EOS by Degryareva et al.^11^ was used for determination of the transition pressure based on Bi.

### Samples: dDAC experiments

Dynamic compression experiments were performed using both the ECB and LLNL dDAC designs^5^, which were used in conjunction with symmetric DACs and LLNL-type membrane DACs, respectively. DACs were equipped with standard design diamond anvils with 200 or 300 μm culets. Stainless steel gaskets were used in most of the experiments, and in a small number of experiments we used WRe gaskets. Data were collected on a total of 14 different samples, and a summary of all experimental runs is given in Table S4. The Bi powder and foil sample materials are the same as those used for the static compression experiments. Au powder was added to all samples to allow us to assess the effect of compression rate on the multi-component sample/pressure marker system, and the pressure was determined based on the position of the (111) Au reflection using the EoS of Au by Fei et al.^26^. For the powder samples, Bi powder was mixed with Au (~ 25 wt%) before loading, whereas for the foil samples loaded with a PTM, a small grain of Au was placed close to the Bi foil so that both could be illuminated by the X-ray beam. For the foil samples loaded without a PTM, Au was placed between the Bi and the diamond culet. The starting pressure of all Ne samples was within the stability field of Bi-III due to either the initial pressure after gas loading being above ~ 2.8 GPa, or the pressure being increased above 2.8 GPa by pre-tightening of the dDAC cap. For the oscillation experiments, all samples were prepared without a PTM and with the Au between the Bi and the diamond anvil.

### X-ray diffraction experiments

X-ray diffraction experiments were performed at the Extreme Conditions Beamline (ECB, P02.2) at PETRA-III^28^, where static and dynamic compression experiments were performed using the same beamline configuration. Data were collected using a 25.7 keV (~ 0.483 Å) incident X-ray beam focused to a ~ 8(h) × 3(v) μm spot using compound refractive lenses. Diffraction images were collected using two LAMBDA 2M detectors that were horizontally offset at either side of the primary X-ray beam at a sample-to-detector (SDD) distance of ~ 420 mm. The SDD, detector tilt and rotation were calibrated based on a Cr_2_O_3_ NIST diffraction standard using the DIOPTAS software^29^. The 2D diffraction images were radially integrated to 1D diffraction profiles using the DIOPTAS software, and data from each detector were analyzed separately and then averaged to determine the pressure and volume/atom for each data point. For the static compression experiments, the lattice parameters for both Bi and Au were determined from a Le Bail fit of the respective structures to the diffraction data using the software JANA2006^30^. In the case of Bi-III, a pseudo-two-phase fit was performed with the constraint that *a*_host_ = *a*_guest_. For the dynamic compression experiments, individual peaks were fit with a Gaussian function for the determination of lattice parameters. The integration of the dDAC into the set-up at the ECB, including information on triggering signals for each component, is described in full in reference^5^. Dynamic compression experiments were performed by applying a trapezoidal voltage waveform with rise times ranging from 25 ms to 1000 s. The detector collection time ranged from 0.5 ms to 1 s, and the number of diffraction patterns collected over the course of a ramp varied from 300 to 6000. The pressure oscillation experiment was performed by applying 60 cycles of a triangular voltage waveform with a frequency of 0.5 Hz to cycle back and forth across the Bi-III/Bi-V phase boundary. For comparison, a further 11 identical samples were compressed with a single triangular voltage waveform with the same rise time.

## Supplementary Information

Supplementary Information.

## Data Availability

The data that support the findings of this study are available from the corresponding author upon reasonable request.
