# Supplementary material for: Compression-rate dependence of pressure-induced phase transitions in Bi
Source: Sci Rep. 2021 Jul 21;11:14859. doi: 10.1038/s41598-021-94260-y (PMC8295338; doi:10.1038/s41598-021-94260-y)
Supplement: Supplementary file 1 — Supplementary Information. [file 41598_2021_94260_MOESM1_ESM.pdf]

Supplementary Information

Compression-rate dependence of pressure-induced phase transitions in bismuth

R. J. Husband et al.

Supplementary methods

Static compression experiments

Static compression experiments were performed on samples loaded in a range of different pressure-transmitting media (PTM) – He, Ne, Ar, and without a PTM. We note that samples compressed in a PTM can only be considered to be truly hydrostatic while the media remains in the fluid phase (below 12.1 GPa for He, 4.8 GPa for Ne, and 1.4 GPa for Ar), but have been reported to remain essentially hydrostatic until higher pressures. Klotz et al. [31] reported that the standard deviation in pressure for Ar, Ne, and He remains below 0.2 GPa up to ~ 18 GPa, indicating that the pressure gradients in our static compression experiments are low. The Ne and He samples are therefore assumed to be hydrostatic. Mixed-phase patterns Bi-III/Bi-V were not observed for samples loaded with a PTM, but were observed for samples loaded without a PTM. When the transition pressure was estimated based on the Au pressure marker, it was therefore taken as the midpoint between the highest-pressure single phase Bi-III pattern and the pattern in which Bi-V was first observed. When the transition pressure was estimated based on Bi, the pattern in which Bi-V was first observed was used.

Data from the foil sample compressed without a PTM, in which the Au was placed between the Bi and one of the diamond anvils, appear to show a higher compressibility than samples loaded with a PTM (Fig. 1). This observation cannot be explained by deviatoric stress in the sample, which would result in a lower compressibility compared to quasi-hydrostatic compression (with a PTM). Instead, the apparent increase in the compressibility of Bi must be related to the behaviour of the pressure marker. When the Au is touching the diamond on one

side but otherwise surrounded by Bi, the resultant stress field and associated strain of the Au is complex. As a result, Bi and Au no longer exist in an isostress condition and indicate different pressures. The apparent increase in Bi compressibility is therefore not a real material property of Bi, but rather an artefact related to the behaviour of the sample and pressure marker in the sample chamber when no PTM is used. In order to determine if this behaviour is unique to Au, this experiment was repeated with Cu as a pressure marker in the same loading configuration (Fig. S1). These data are in good agreement with those from Au, suggesting that this behaviour is not unique to Au.

Experiments were performed to investigate the influence of the location of Au within the sample chamber on the measured compression curves (Fig. S1). When the Au is embedded between two pieces of Bi foil so that it does not touch the diamonds, the data indicate a lower compressibility than the data collected from samples loaded with a PTM. In this case, Bi itself acts as a PTM and minimizes the uniaxial stress in Au, and the (upward) shift in the compression curve is most likely related to deviatoric stress in the Bi. Data collected from a Bi powder sample mixed with Au powder lie between those from the embedded Au sample and data from samples loaded in He and Ne. However, for the powder Bi/Au sample, the stress field of different Au grains can be quite different depending on whether they are in contact with the diamond, Bi, or other grains of Au. This loading configuration is therefore not a reliable method of pressure determination. Based on our observations on samples loaded without a PTM, it may be that the results from the previous study by Akahama et al. [21] were influenced by their use of stacked foils of Bi, Au and Pt; they found that the pressure determined from Au was systematically lower than that determined by Pt, although the discrepancy was not significant until higher pressures than those considered here. In the future, it may be possible to avoid these issues by choosing a pressure marker with similar elastic moduli to the sample material.

When comparing the behaviour of samples loaded with and without a PTM, the stress state of the sample should be considered. Deviatoric stress can be identified based on shifts of Bragg reflections from their expected positions [32]. As we have identified the Bi-III/Bi-V transition by on the onset pressure, it is therefore necessary to consider the stress state of Bi-III. Le Bail refinements of the Bi-III structure based on diffraction patterns collected from samples loaded without a PTM show evidence of line shifts that are not observed for patterns collected from samples loaded in Ne (Fig. S6), suggesting a higher degree of deviatoric stress in samples loaded without a PTM. Although it would be beneficial to determine the uniaxial stress component using (for example) a line shift analysis [32], this is unfortunately not possible due to the complex nature of the host-guest structure. Although it is in principle possible to perform a line-shift analysis for Bi-V, this would not be representative of the stress state before the transition because of the volume drop at the transition (~1 %), which could allow for partial stress release.

dDAC experiments

Samples were compressed by applying a trapezoidal voltage waveform as described in the main paper. Different dDAC designs (ECB and LLNL) employ different piezo actuators, and so DACs compressed using the different designs will respond differently to the same applied voltage. As is typical for compression ramps using both dDAC designs, the pressure does not have a linear response to the applied voltage and compression rate is not constant throughout the ramp, as can be seen in Fig. 2. Typically, the sample pressure does not increase until ~100V has been applied to the piezo actuator and the compression rate is much higher at later times in the ramp. This is most likely due to the mechanical response of the DAC, piezo and dDAC assembly and is independent of the sample material. For this reason, we have chosen to use the instantaneous compression rate and not the average compression rate when comparing different sample runs. For the instantaneous compression rate, the average rate over

the last 5-10 single-phase Bi-III patterns before the transition was used, where lower-pressure patterns were chosen due to the definition of the transition by the onset pressure and to avoid the effect of the (small) volume change associated with the Bi-III/Bi-V transition. The kink at ~5 GPa in the slow compression profile is most likely associated with the solidification of Ne. For the slow ramps, the pressure at the top of the ramp as estimated from the two detectors can differ by as much as 0.5 GPa which is evidence of an asymmetric radial stress field in the sample chamber. Note that the detectors are symmetrically offset in the horizontal, but they are translated down in the vertical so that they collect the upper portion of the diffraction rings. Although stress gradients of this magnitude in the Ne loaded sample appear surprising, it should be noted that Ne is not truly hydrostatic at the pressures > 4.8 GPa as it is a solid. On release, the sample pressure often did not fully decompress to the starting pressure. This was consistently more pronounced for hydrostatic sample loadings, and in many cases the pressure had to be released my loosening the screws of the DAC for the sample to transform back to Bi-III. This effect was observed in all experimental runs and is not sample dependent, but most likely due to plastic deformation of the metallic gasket.

For samples loaded without a PTM, the same sample could be compressed through the Bi-III/Bi-V transition up to 8 times at different compression rate. For samples loaded in a PTM, the number of compression cycles per sample loading was on the order of 1-5 times since the PTM can escape out of the sample chamber upon rapid decompression possibly resulting in a loss of PTM. The loss of the PTM was carefully monitored from the absorption scans performed to align the sample on the beamline, and occasionally also by examining the sample under the microscope. The pressure during the compression cycle was determined based on a fit of the Au (111) reflection using a Gaussian function. For analysis of the compression curves, the unit cell volume of Bi-III was determined from the peak positions of three reflections for consistency: (2110), (3100) and (2101), as these were clearly observed for all samples. The (hklm) superspace notation for Bi-III is described by McMahon et al. [33] The unit cell volume of

Bi-V was determined from the peak position of the (211) reflection in the mixed phase patterns, and from both the (110) and (211) positions at higher pressures.

As the samples were compressed through the Bi-III/Bi-V transition, significant changes in microstructure were observed for both sample types; the diffraction patterns from Bi-III were consistently observed to be much spottier than those from Bi-V (Fig. S8). Due to the spotty nature of the Bi-III diffraction images, the Bi-III/Bi-V phase transition was identified by the first observation of Bi-V in the diffraction patterns. The (110) and (200) Bi-V reflections both overlap with peaks from the Bi-III phase, and so in most cases the onset of the transition was identified by the appearance of the (211) Bi-V reflection. When Au was used for pressure estimation, the onset transition pressure was taken as the midpoint between that of the last single-phase Bi-III pattern and the first mixed-phase Bi-III/Bi-V pattern. When the pressure was determined from Bi-V, the onset transition pressure was determined as the pressure of the pattern in which Bi-V is first observed. Although it would be desirable to also determine the pressure using the EOS of Bi-III, this was not always possible due to the pronounced preferred orientation in this phase; Bi-III reflections were not consistently observed in all experimental runs, and in many cases there were not enough observed reflections to determine all three lattice parameters (a, c_{host} and c_{guest}). Due to the spotty nature of the diffraction patterns from the foil sample and the fact that the detectors do not give full azimuthal coverage and diffraction spots that originate in areas between the sensitive areas of the detectors cannot be detected, we note that the reported transition pressures for the foil samples likely do not represent the lowest pressure where Bi-V is observed but some higher pressure that is still below the pressure at which the Bi-V phase transformation is fully completed.

dDAC Experiments: Sample/pressure marker assembly

We compared the transition pressures determined based on Bi and Au for all dynamic compression experiments (Fig. S4). For samples loaded in Ne, transition pressures determined

using both methods are in good agreement (Fig S4a,b), suggesting that the use of a pressure marker for samples loaded in a ‘soft’ pressure medium like Ne is justified. However, for samples loaded without a PTM, the compression-rate response is highly dependent on the position of the pressure marker within the sample chamber. For mixed Bi/Au powder samples compressed without a PTM, the Au-determined transition pressures are very scattered, and no clear compression-rate dependent shift is evident (Fig. S4c). For foil samples loaded without a PTM, the magnitude of the Bi-III/Bi-V over-pressurization is overestimated by the Au (Fig. S4d). A compression-rate dependent response of the Bi/Au assembly is clearly visible in the compression curves collected during fast and slow compression (Fig. S2), and in the integrated diffraction patterns collected at the same pressure during fast and slow compressions (Fig. S3 and Table S3). These results highlight a potential pitfall for future dDAC experiments, where sample/pressure marker effects can potentially be incorrectly identified as compression-rate dependent effects in the sample.

Supplementary Figures

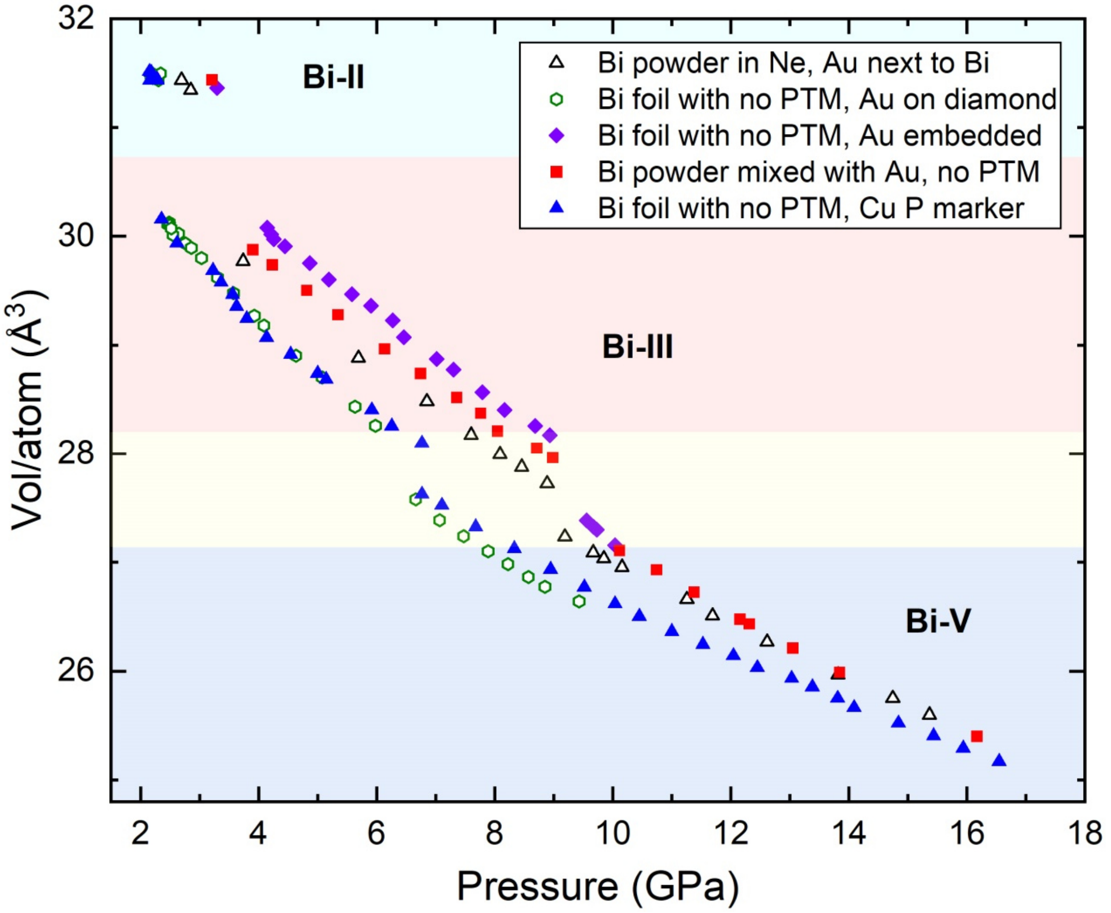

Fig. S1. Volume/atom of Bi as a function of pressure for additional static compression experiments. Data are shown for a Bi foil sample loaded with a Cu pressure marker placed between the Bi and the diamond, a Bi foil sample with the Au pressure marker sandwiched between two pieces of Bi foil, and a sample of Bi powder mixed with 20% Au powder. These are compared with data from the Bi powder sample in Ne and the Bi foil sample without a PTM from in Fig. 1, both of which used an Au pressure marker. The reported transition pressures are estimated from Au.

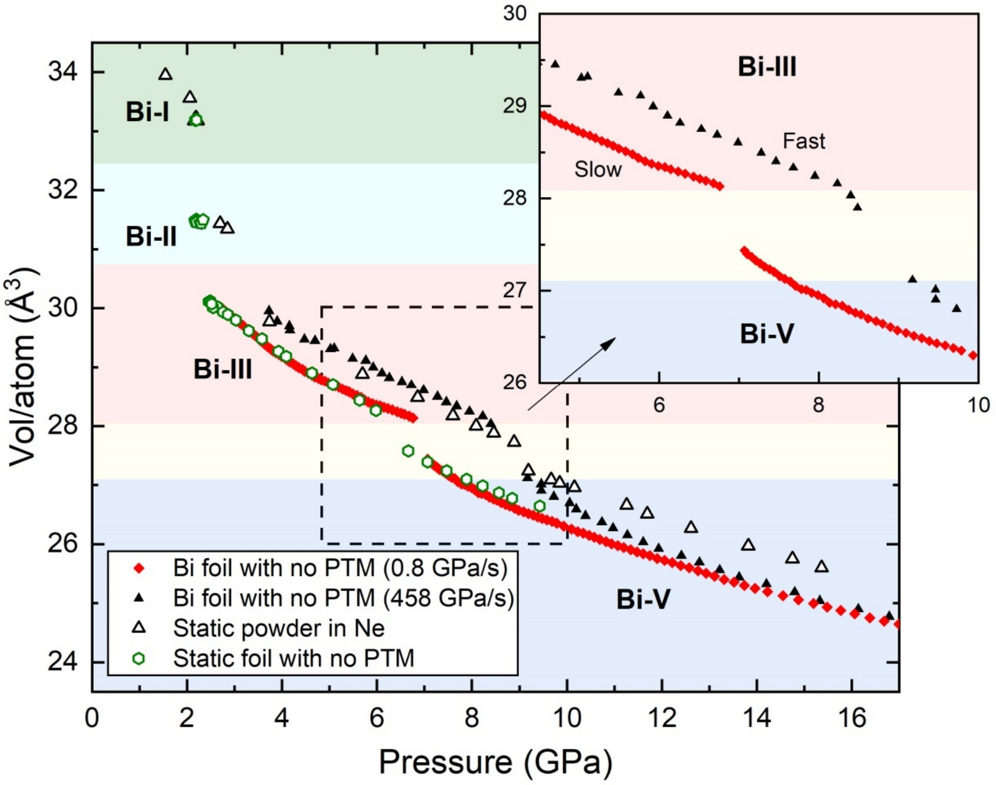

Fig. S2. Volume/atom as a function of pressure for fast and slow dDAC compression ramps on Bi foil samples loaded without a PTM. These data are compared with the results of static compression experiment on the Ne powder sample from Fig. 1. Samples were prepared with the Au placed between the Bi and the diamond, and the pressure was determined from the Au pressure standard as described in the text. The inset highlights the discrepancy between the fast and slow compression curves in the vicinity of the Bi-III/Bi-V phase transition, which is due to the different compression-rate response of Bi and Au in this loading configuration.

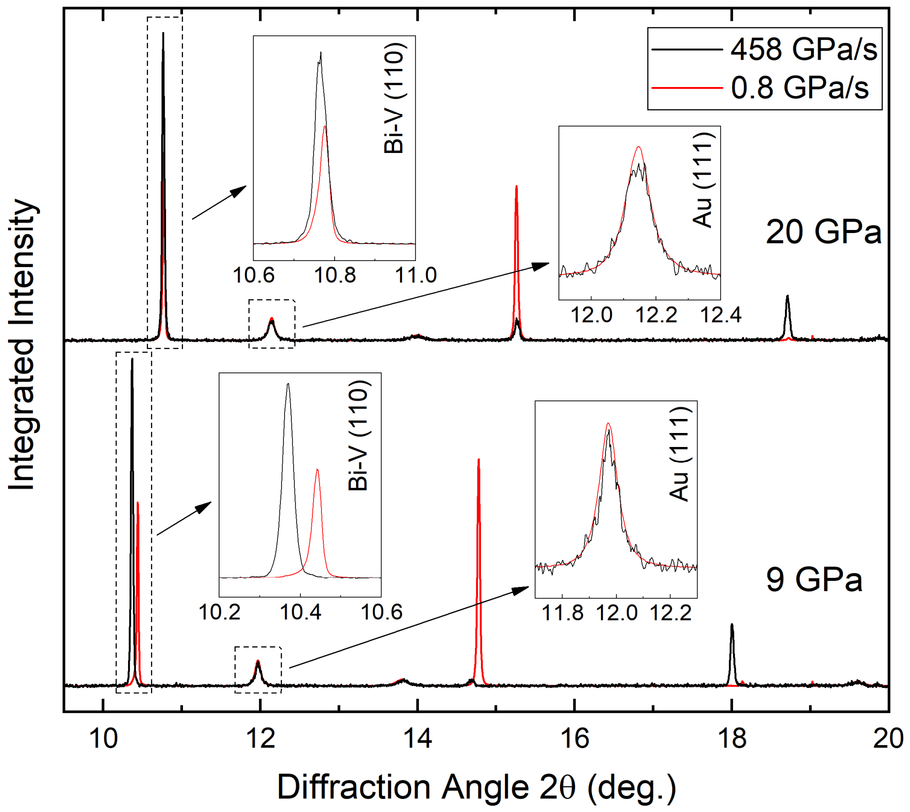

Fig. S3. Comparison of integrated X-ray diffraction profiles at collected during fast and slow compressions of Bi foil samples loaded without a PTM, where the corresponding compression curves are shown in Fig. S2. When the Au reflections in the two ramps are observed at the same *d*-spacing during compression, the Bi reflections are observed are at higher angles (lower *d*-spacings) during slow compression than those observed during fast compression. This suggests that for this specific loading configuration (Au placed between the Bi and the diamond anvil), the relative pressures experienced by the Bi and Au can differ depending on the compression rate.

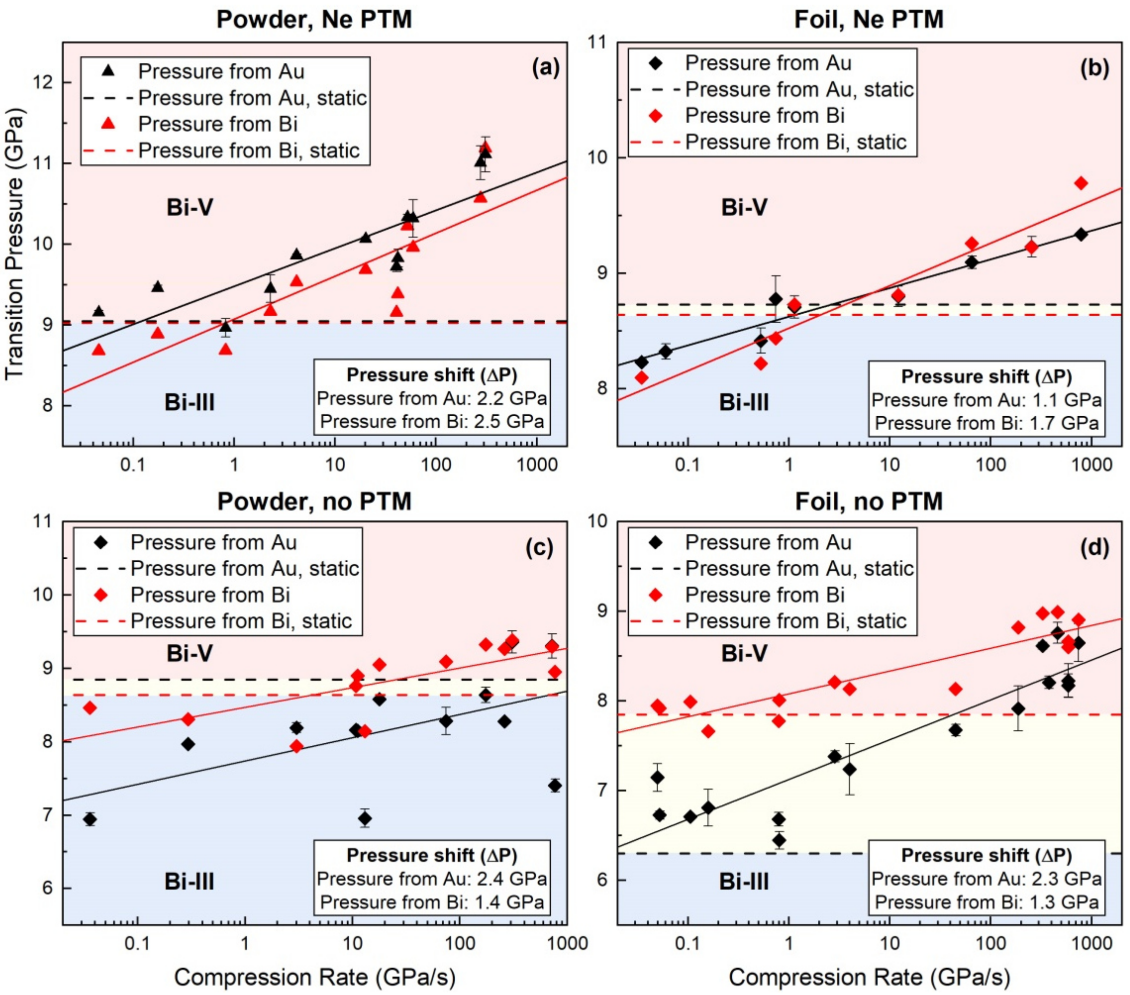

Fig. S4. Comparison of the Bi-III/Bi-V transition pressure determined from Au and Bi as a function of compression rate. **(a)** and **(c)** show data from powder samples loaded in Ne and without a PTM, respectively, and **(b)** and **(d)** show data from foil samples loaded in Ne and without a PTM, respectively. When the pressure is determined from Au, the error bars are based either on the pressure difference between subsequent diffraction patterns or from the difference in the pressure determined from each of the detectors, where the larger value was used. Although the same method cannot be used in the case of Bi, the error bars are assumed to be of the same magnitude as when Au is used. The solid lines show a fit of the dynamic compression data to functional form $y = Alog(x) + B$, and the resultant fit parameters are given in Table S3.

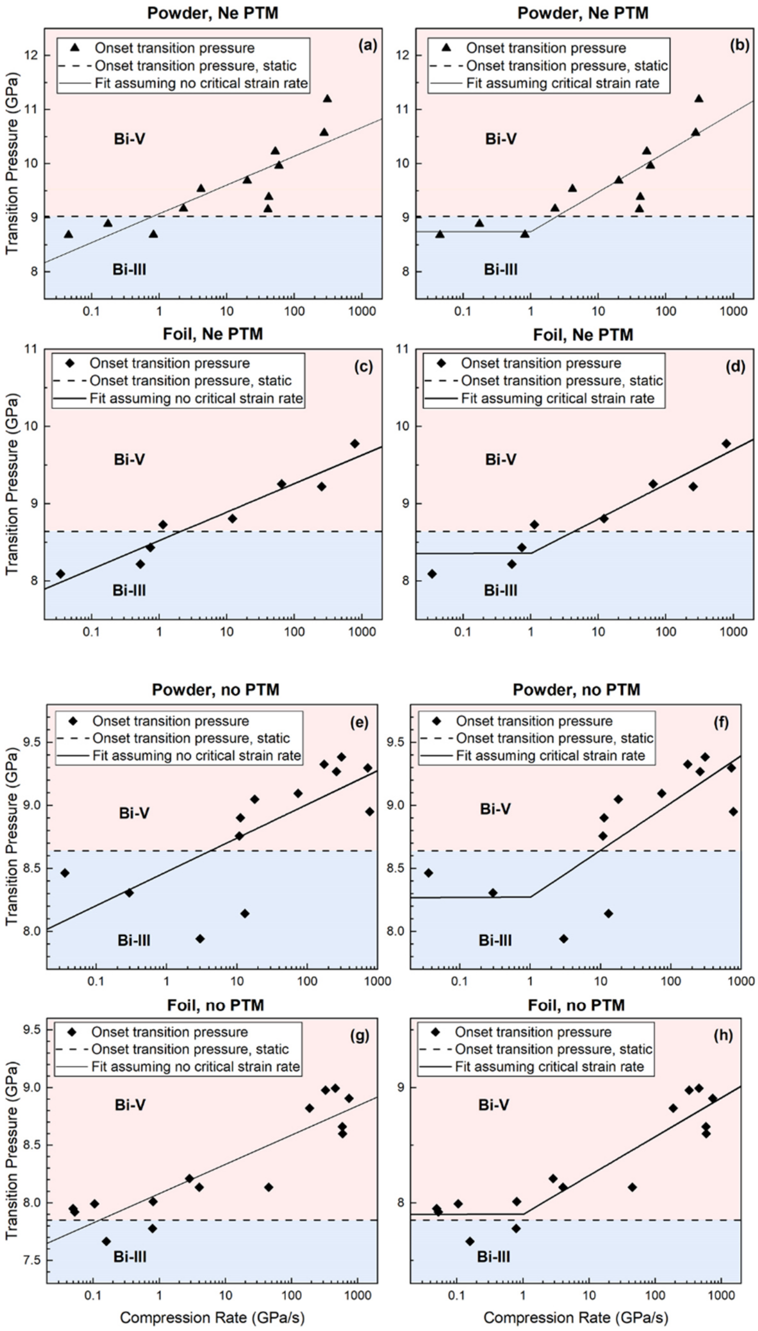

Fig. S5. Comparison of different fits to the Bi-III/Bi-V transition pressure data collected during dynamic compression experiments for different sample types (powder and foil) and different stress states (with and without a PTM). Panels (a), (c), (e) and (g) show fits to the functional form $y = A\log(x) + B$. Panels (b), (d), (f) and (h) show fits of the same data assuming a critical compression rate of 1 GPa/s.

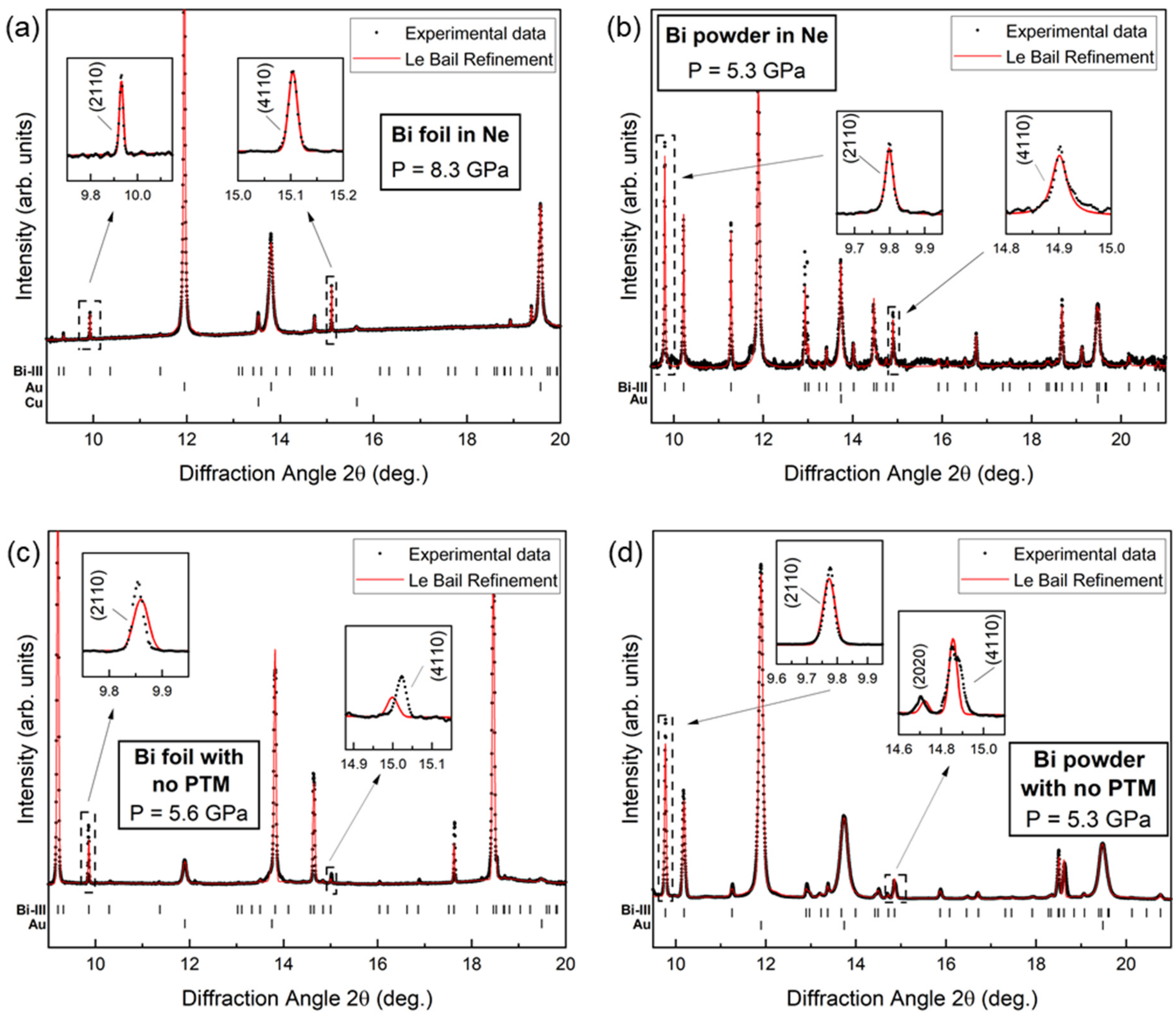

Fig. S6. Le Bail refinements of the Bi-III structure based on diffraction patterns collected from (a) Bi foil in Ne at 8.3 GPa, (b) Bi powder in Ne at 5.3 GPa, (c) Bi foil without a PTM at 5.6 GPa and (d) Bi powder without a PTM at 5.3 GPa. The black points show experimental data, the red solid line shows the fit, and the tick marks below the pattern show the calculated peak positions. The inserts highlight the quality of the fit to the (2110) and (4110) reflections. Although an excellent fit to the (4110) reflection is observed for samples loaded in Ne, a poor fit is observed for samples loaded without a PTM.

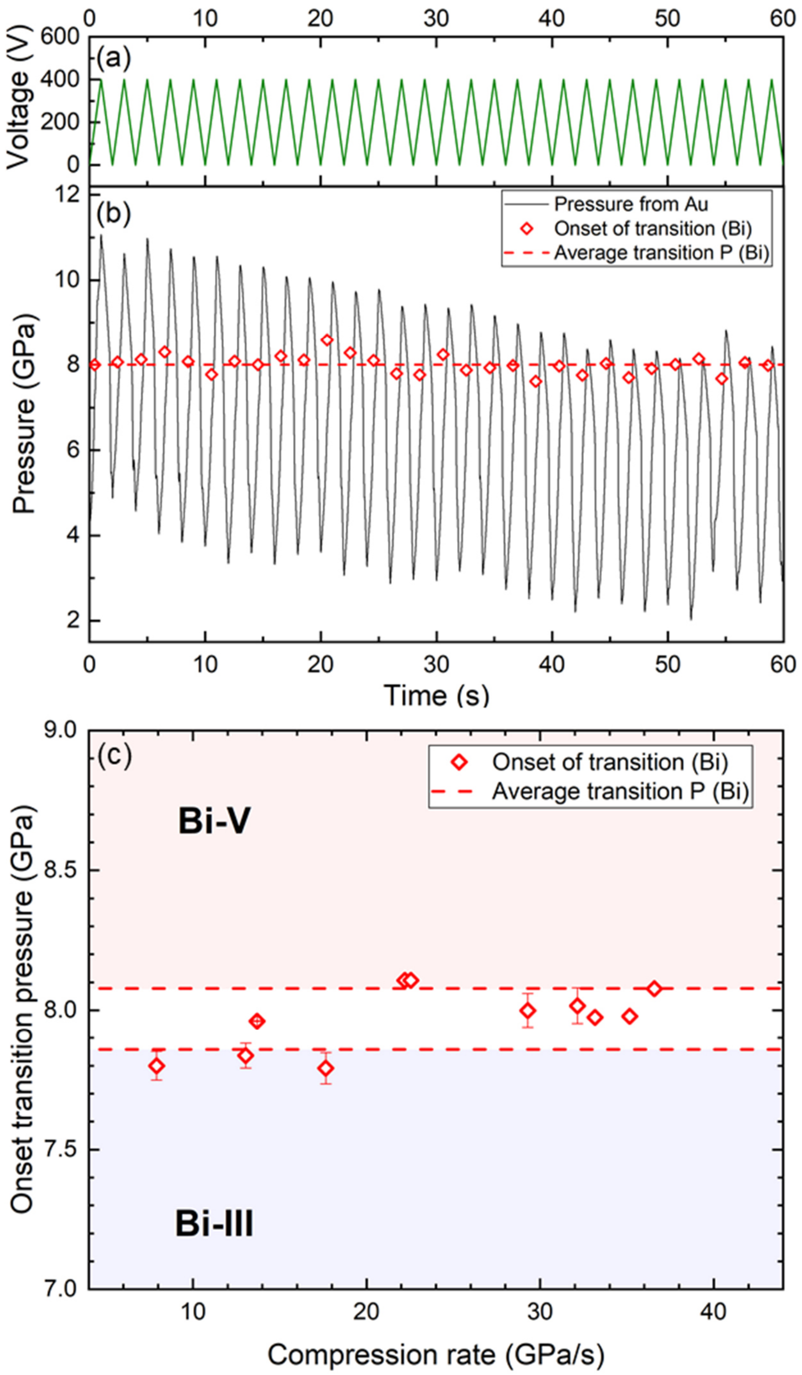

Fig. S7. (a) Time-dependent pressure profile of a Bi powder sample compressed 60 times through the Bi-III/Bi-V transition, where the pressure was determined from Au. The time-dependent voltage applied to the piezo-actuator is shown in (b). The red points show the onset Bi-III/Bi-V transition pressure determined from the unit cell volume of Bi-V, and the average value is indicated by the dotted red line.

(c) Bi-III/Bi-V transition pressure for a set of newly-loaded Bi powder samples. Each sample was compressed with a single triangular voltage waveform with a 0.5 s rise time. The red diamonds show the transition pressure determined from the unit cell volume of Bi-V, where the error bars were determined from the difference in pressure estimated from different detectors. In some runs data were collected using only one detector and so the error bars are omitted. The dotted lines indicate the region of uncertainty of the average transition pressure from all samples.

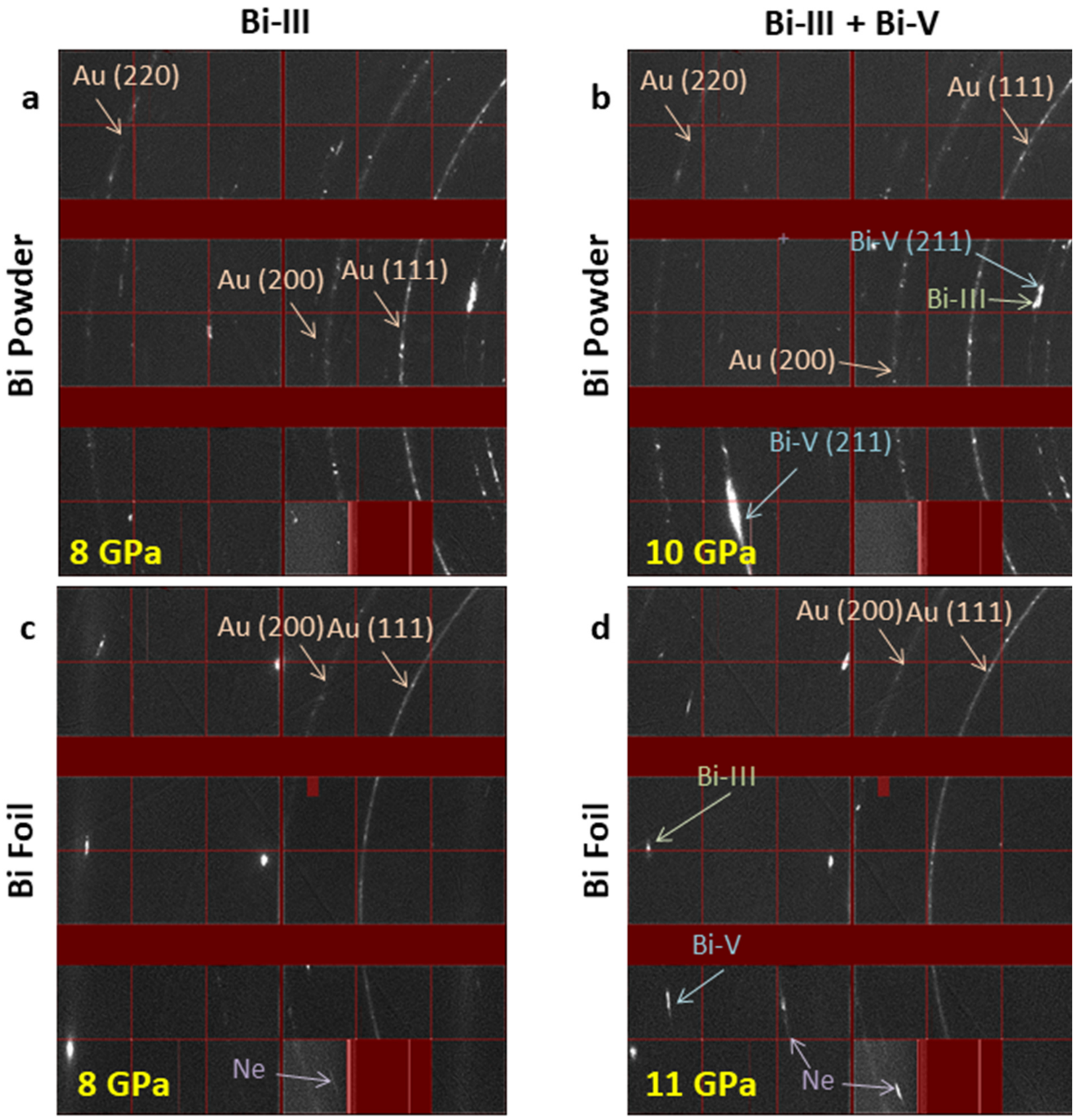

Fig S8. 2D diffraction images collected from one of the LAMBDA 2M detectors during the compression of powder and foil samples loaded in Ne. The diffraction images were collected from the powder sample in the (a) Bi-III and (b) mixed Bi-III/Bi-V phases, and the foil sample in the (c) Bi-III and (d) mixed Bi-III/Bi-V phases. The collection time was 1s. The red, masked area is not included in the integration and corresponds to gaps between detector modules. All reflections from Au, Bi-V and Ne are labelled; all other reflections are from Bi-III.

Supplementary tables

Table S1. Bi-III/Bi-V transition pressures for samples compressed without a PTM, where the pressure marker was placed in different locations in the sample chamber.

**Sample Type**	**Pressure marker**	**Position in sample chamber**	**Pressure determinant**	**Transition Pressure (GPa)**
Foil	Au	Between Bi and diamond	Au	6.3(4)
			Bi	7.86
Foil	Au	Embedded	Au	9.2(2)
			Bi	8.21
Powder	Au	Mixed	Au	8.85(14)
			Bi	8.86
Foil	Cu	Between Bi and diamond	Au	6.94(17)
			Bi	7.71

Table S2. Fit parameters from Fig. 4 and Fig. S4, where the dynamic compression data were fit to the functional form $y = Alog(x) + B$.

Sample Type	Pressure Transmitting Medium (PTM)	Pressure Determination Method	Fit parameter A (GPa)	Fit parameter B (GPa)	R^2
**Powder**	Ne	Bi	0.53(11)	9.07(16)	0.71
**Powder**	No PTM	Bi	0.27(8)	8.47(14)	0.53
**Foil**	Ne	Bi	0.37(4)	8.52(7)	0.92
**Foil**	No PTM	Bi	0.25(3)	8.08(6)	0.81
**Powder**	Ne	Au	0.47(9)	9.48(13)	0.74
**Powder**	No PTM	Au	0.32(14)	7.7(3)	0.31
**Foil**	Ne	Au	0.25(2)	8.63(4)	0.95
**Foil**	No PTM	Au	0.44(5)	7.13(10)	0.92

Table S3. Lattice parameters of Bi and Au for the data in Fig. S3.

The lattice parameters a_{au} and a_{bi} are calculated for all observed Bragg reflections.

	**Pressure (GPa)**			
	9		20	
	**Compression Rate (GPa/s)**			
	0.8	458	0.8	458
**a_{au} (111) (Å)**	4.0131(2)	4.0123(2)	3.9561(2)	3.9562(3)
**a_{au} (200) (Å)**	4.0222(10)	4.0162(10)	3.9661(14)	3.9686(2)
**a_{au} (220) (Å)**	4.0167(11)	4.0165(10)	3.961(2)	3.9588(16)
**$\bar{a}_{au}$ (Å)**	4.017(6)	4.015(2)	3.961(7)	3.961(9)
**a_{bi} (110) (Å)**	3.755200(16)	3.781029(9)	3.639341(16)	3.642159(12)
**a_{bi} (200) (Å)**	3.756780(11)	3.7807(6)	3.638923(16)	3.63725(18)
**a_{bi} (211) (Å)**	3.7545(4)	3.78186(3)	3.6380(10)	3.64041(6)
**$\bar{a}_{bi}$ (Å)**	3.7555(16)	3.7812(8)	3.6387(10)	3.640(4)

Table S4. Summary of all dDAC experimental runs.

Sample number	Bi type	PTM	Run no.	Starting pressure (GPa)	Maximum pressure (GPa)	Rise Time (s)	Average compression rate (GPa/s)	Instantaneous compression rate (GPa/s)
1	Foil	Ne	1	3.0	19.2	0.3	54	65
			2	4.2	9.6	0.05	108	255
			3	3.0	19.7	300	0.06	0.06
2	Foil	Ne	1	2.8	19.8	0.025	680	785
			2	4.1	8.5	30	0.18	0.5
			3	4.4	26.4	2	11	12
			4	4.6	14.7	900	0.011	0.015
3	Foil	Ne	1	5.5	15.8	30	0.34	0.74
4	Foil	Ne	1	4.3	21.2	30	0.56	1.13
5	Foil	Ne	1	4.2	37.5	1000	0.033	0.035
6	Foil	No PTM	1	2.1	21.2	0.05	382	458
			2	1.3	12.7	0.025	456	741
			3	0.1	12	5	2.4	4
			4	0.5	21.1	30	0.69	0.80
			5	1.4	20.7	30	0.64	0.79
			6	0.5	20.2	300	0.066	0.16
7	Foil	No PTM	1	2.4	15.3	250	0.052	0.10
8	Foil	No PTM	1	1.8	16.2	0.1	144	187
			2	1.2	17.1	0.05	318	378
			3	2.5	18.6	0.05	322	326
			4	2.4	18.6	0.025	648	587
			5	4.1	23.7	5	3.9	2.8
			6	0.8	10.6	300	0.033	0.05
			7	0.6	8.7	0.3	27	45
			8	0.6	9.0	0.025	336	590

9	Foil	No PTM	1	0	11.3	600	0.019	0.05
10	Powder	Ne	1	4	14.6	0.5	21	42
			2	5.5	22.5	10	1.7	2.3
			3	4.2	9.9	20	0.29	0.8
			4	5.7	14.7	0.2	45	41
11	Powder	Ne	1	5.4	12.8	0.15	49	52
			2	9.1	4.2	11.1	68	309
			3	4.3	26	0.15	145	277
12	Powder	Ne	1	2.1	12.8	0.5	21	20
			2	3.8	21.4	0.5	35	59
			3	3.6	20.9	5	3.5	4.16
			4	7.6	15.8	60	0.14	0.17
			5	3.8	11.6	300	0.03	0.05
13	Powder	No PTM	1	0	13.3	2	6.7	18
			2	1.5	9.1	0.3	25	74
			3	2.3	11.9	0.1	96	174
			4	3.3	11.8	300	0.03	0.04
			5	3.3	9.7	1	6.4	13
			6	2.8	9	0.03	207	772
14	Powder	No PTM	1	2.6	22.5	0.05	398	722
			2	4.1	24.2	0.05	402	307
			3	4.8	16.8	1	12	11
			4	4.1	17.9	1	14	11
			5	4.3	18.1	5	2.8	3
			6	4.3	16.3	0.05	240	261
			7	4.2	19.1	60	0.2	0.3

Supplementary References

31 Klotz, S., Chervin, J. C., Munsch, P. & Le Marchand, G. Hydrostatic limits of 11 pressure transmitting media. *Journal of Physics D-Applied Physics* **42**, 075413, doi:10.1088/0022-3727/42/7/075413 (2009).

32 Singh, A. K. & Balasingh, C. Analysis of lattice strains measured under nonhydrostatic pressure. *Journal of Applied Physics* **83**, 7567, doi:10.1063/1.367872 (1998).

33 M. I. McMahon, M. I., Degtyareva, O., Nelmes, R. J., van Smaalen, & S., Palatinus, L. Incommensurate modulations of Bi-III and Sb-II. *Physical Review B* **75**, 184114, doi:10.1103/PhysRevB.75.184114 (2007).
